# Performances of birthweight charts to predict adverse perinatal outcomes related to SGA in a cohort of nulliparas

**DOI:** 10.1186/s12884-022-04943-1

**Published:** 2022-08-04

**Authors:** Rafael B. Galvão, Renato T. Souza, Matias C. Vieira, Dharmintra Pasupathy, Jussara Mayrink, Francisco E. Feitosa, Edilberto A Rocha Filho, Débora F. Leite, Janete Vettorazzi, Iracema M. Calderon, Maria H. Sousa, Jose G. Cecatti, for the Preterm SAMBA study group

**Affiliations:** 1 Department of Obstetrics and Gynaecology, University of Campinas (UNICAMP), Campinas, Brazil; 2 Department of Women and Children’s Health, King’s College London, London, UK; 3 Specialty of Obstetrics, Gynaecology and Neonatology, Westmead Clinical School, University of Sydney, Sydney, Australia; 4 School Maternity, Federal University of Ceará (MEAC), Fortaleza, Brazil; 5 Department of Maternal and Child Health, Federal University of Pernambuco, Recife, Brazil; 6 Department of Obstetrics and Gynaecology, Maternity of the Clinic Hospital, Federal University of RS, Porto Alegre, Brazil; 7 Department of Gynaecology and Obstetrics, Botucatu Medical School (Unesp), Botucatu, Brazil; 8 Statistics Unit, Jundiai School of Medicine, Jundiaí, Brazil

**Keywords:** Small-for-gestational-age, Fetal growth restriction, Nulliparity, Neonatal morbidity, Adverse neonatal outcome, Birthweight, Birthweight chart, Birthweight centiles

## Abstract

**Background:**

Small-for-gestational-age neonates (SGA) are at increased risk of neonatal morbidity. Nulliparity represents a risk factor for SGA; birthweight charts may perform differently for the detection of SGA among nulliparas. This study aimed at describing the prevalence of SGA in nulliparas according to different birthweight charts and evaluating the diagnostic performance of these charts to maternal and perinatal outcomes.

**Methods:**

This is a secondary analysis of a Brazilian cohort of nulliparas named Preterm SAMBA study. Birthweight centiles were calculated using the Intergrowth-21st, WHO-Fetal Growth Charts, Birth in Brazil population chart and GROW-customised chart. The risks of outcomes among SGA neonates and their mothers in comparison to neonates with birthweights between the 40^th^-60^th^ centiles were calculated, according to each chart. ROC curves were used to detect neonatal morbidity in neonates with birth weights below different cutoff centiles for each chart.

**Results:**

A sample of 997 nulliparas was assessed. The rate of SGA infants varied between 7.0–11.6%. All charts showed a significantly lower risk of caesarean sections in women delivering SGA neonates compared to those delivering adequate-for-gestational-age neonates (OR 0.55–0.64, *p* < .05). The charts had poor performance (AUC 0.492 – 0.522) for the detection of neonatal morbidity related to SGA born at term.

**Conclusion:**

The populational and customised birthweight charts detected different prevalence of small-for-gestational-age neonates and showed similar and poor performance to identify related neonatal adverse outcomes in this population.

**Supplementary Information:**

The online version contains supplementary material available at 10.1186/s12884-022-04943-1.

## Background

Neonates classified as small-for-gestational-age (SGA) can be constitutionally small individuals exhibiting physiological growth or result from pathological pregnancies that became growth-restricted (unable to achieve their growth potential). These SGA neonates are often associated with a higher risk of neonatal morbidity, and perinatal mortality, in addition to long-term morbidity such as chronic conditions that persist throughout adult life [1–3]. Many factors are known to be associated with an increased risk of SGA, such as nulliparity, malnutrition, extremes of maternal age, and tobacco and/or drug use, among others. According to a study based on a large Brazilian population, nulliparity was a significant risk factor for SGA (OR 1.81, 95%CI: 1.60–2.05) with a population attributable fraction of 24%, meaning that 24% of SGA could be attributed to nulliparity [4].

Identifying SGA neonates who are at a higher risk of adverse outcomes is of the utmost importance to increase surveillance actions toward the neonate and to prevent related neonatal morbidity [5–7]. Most importantly, appropriate classification of birthweight abnormalities plays a key role in the accurate identification of SGA and is fundamental for proper management.

There are different methods for calculating birthweight centiles, which may vary according to their developing method. International birthweight standards, such as the Intergrowth-21^st^ study charts and the WHO-Fetal Growth Charts study, are based on a ‘healthy’ sample composed of well-nourished pregnant women, without important risk factors for fetal growth abnormalities [8, 9]. The birth weight chart from the Birth in Brazil study is a population standard developed and based on a retrospective analysis that excluded high-risk pregnancies from a large cohort recruited through a complex sampling strategy to represent the Brazilian population [10]. In addition, there are customised growth charts that use a pregnancy-related optimal weight (GROW) software [11], adjusting not only for neonatal but also maternal characteristics including age, maternal weight, maternal height, parity and ethnicity to provide an individual assessment of fetal growth [12].

The diagnostic performance of a growth curve may vary according to the characteristics of the population to which it is applied [13, 14]. Considering that nulliparas are at a higher risk for SGA neonates, thus for neonates with a higher risk of adverse outcomes, the performance of different birthweight charts in the diagnosis of neonatal morbidity in SGA infants needs to be evaluated in such a high-risk population.

The current study aimed to describe the prevalence of SGA in nulliparous women according to different birthweight charts from the INTERGROWTH-21^st^ Project [9], the birth weight chart from World Health Organization—Fetal Growth Charts (WHO-FGC) [8], the Birth in Brazil population chart [4] and the GROW-customised growth chart [12] and evaluate the diagnostic performance of these charts to identify adverse perinatal outcomes using different thresholds to define small infants using a cohort of nulliparous women.

## Methods

This is a secondary analysis of the Preterm SAMBA Study, a prospective multicenter cohort study that aimed at evaluating metabolomic profiles, associated with adverse outcomes in women delivering singleton pregnancies from five participating centres in Brazil, from July 2015 to July 2018 [15]. The Preterm SAMBA study enrolled 1,373 nulliparous pregnant women followed from 19–21 weeks of gestational age until childbirth. The study gathered maternal and neonatal data to evaluate the incidence of SGA neonates and its association with adverse neonatal outcomes. The study was approved by the local Institutional Review Board from each participating centre (CAAE: 38,522,214.8.1001.5404) and all participants signed an informed consent form before study admission. Methodological details and related procedures of the Preterm SAMBA Study have been detailed in previous publications elsewhere [15, 16].

Briefly, in the Preterm SAMBA study, data collection was based on three study visits during pregnancy and a medical record review for the information on late pregnancy, delivery, and post-partum. At the first visit (19–21 weeks) sociodemographic data and medical history were collected, along with clinical data and evaluation of dietary habits and anthropometric measures. At the second and third visits (27–29 weeks and 37–39 weeks, respectively), the same clinical data were collected. Finally, a review of the medical records from the women and their newborns was performed to collect data on late pregnancy, childbirth, postpartum and neonatal aspects. During the Preterm SAMBA study, standard procedures and definitions were applied to major outcomes such as preterm birth [17], preeclampsia [18], gestational diabetes mellitus [19] and SGA [17].

For the current secondary analysis, women with sufficient data to calculate birthweight centiles using all the birthweight charts. Preterm birth is a pathological manifestation per se and prematurity represents a confounding factor in the assessment of adverse perinatal outcomes. Therefore, only those who had delivered term infants (gestational age ≥ 37 weeks) were included. We excluded neonates with any major anatomic congenital abnormality.

Birthweight centiles were calculated using international population charts such as the birthweight chart from Intergrowth-21^st^, the birthweight chart from WHO-Fetal Growth Charts (WHO-FGC) [8, 9], the Birth in Brazil population chart [4] and the GROW customised birthweight chart (GROW) [11]. Assuming the representativeness of the Brazilian population, the Birth in Brazil chart was considered the reference method for comparison. The standard cutoff point used for detecting SGA neonates was the traditionally adopted 10^th^ centile for all charts.

For birthweights below the 10^th^ centile in each chart, we calculated the rate of maternal and neonatal outcomes, such as overall caesarean section rates, need for neonatal intensive care unit (NICU) admission, low 5-min Apgar score (< 7), perinatal mortality (stillbirth or early neonatal death) and neonatal morbidity, which was defined as a composite outcome based on the occurrence of hypoxic-ischemic SGA related neonatal outcomes, according to previously published reports [20]. Therefore, neonatal morbidity was considered if the neonate presented at least one of the following: hypoxic-related conditions, such as hypoglycemia, seizures, persistent pulmonary hypertension, bronchopulmonary dysplasia, hypoxic-ischemic encephalopathy, intraventricular haemorrhage, necrotizing enterocolitis, sepsis or use of mechanical ventilation or oxygen therapy. In addition, the odds ratios and their 95% confidence intervals (CI) for each outcome were calculated considering the reference group for adequate-for-gestational-age, between the 40^th^ and 60^th^ centiles, infants who are presumed to be at the lowest risk for adverse outcomes [13].

We compared the diagnostic performance for detecting neonatal morbidity of all previously selected birthweight charts. We calculated the detection rate, sensitivity, false-positive rate and the area under the ROC curve for different centile thresholds (birth weight below 5^th^, 10^th^, 15^th^ and 20^th^ centiles) for each chart.

All analyses and calculations of birthweight centiles according to different charts were performed using IBM SPSS® Statistics v.20 and Stata® v.12. Variables were tested for normality distribution and statistical tests were applied accordingly.

## Results

**Fig. 1:**
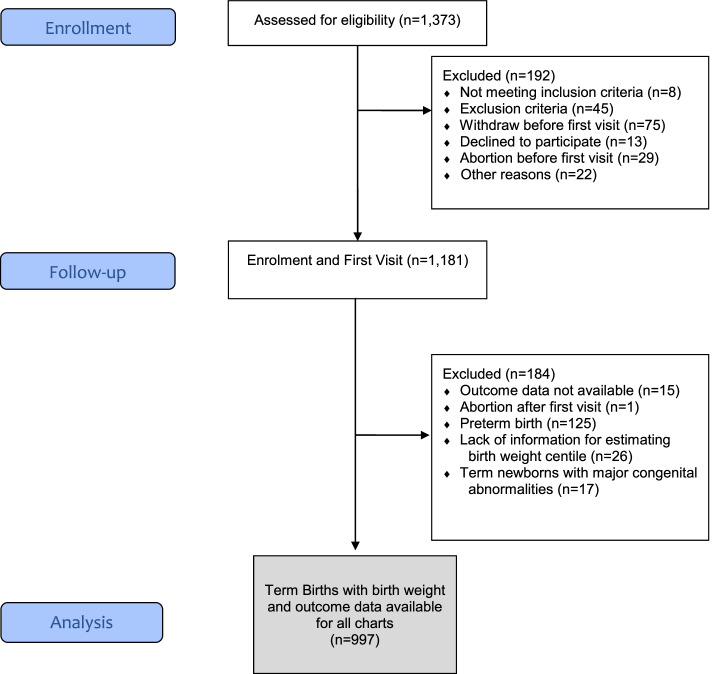
Study population

A total of 1,373 women were enrolled in the Preterm SAMBA Study. Of these, 997 (72.6%) were included in the current analysis (Fig. 1). The characteristics of the study population and the frequency of maternal and perinatal outcomes are shown in Tables 1 and 2. The majority of the participants were non-white women, aged between 20 and 34 years, with a partner, in paid work, with less than 12 years of schooling and of middle-class family income. The mean gestational age at delivery was 39.0 (± 1.1) weeks and neonates had a mean birth weight of 3,253 g (± 419). Neonatal morbidity occurred in 35 (3.6%) neonates, eight (0.8%) of which had low 5-min Apgar scores and seven (0.7%) had hypoglycemia. A total of 64 (6.4%) and 127 (12.7%) women developed preeclampsia and hyperglycemia in pregnancy after enrolment in the study.

**Table 1:** Demographic characteristics of the included Preterm SAMBA population

Characteristics	Study population N (%)
Region	
Northeast	476 (47.7)
South and Southeast	521 (52.3)
Maternal age (years)	
≤ 19	252 (25.3)
20–34	680 (68.2)
≥ 35	65 (6.5)
Ethnicity	
White	405 (40.6)
Non-white	592 (59.4)
Marital status	
With partner	733 (73.5)
Without partner	264 (26.5)
Maternal Occupation	
Paid work	493 (49.4)
Homemaker	182 (18.3)
Not working	322 (32.3)
Schooling (years)	
< 12	673 (67.5)
≥ 12	324 (32.5)
Annual Family Income (US$)	
Up to 3,000	45 (4.5)
3,000 to 12,000	537 (53.9)
Above 12,000	415 (41.6)
Source of prenatal care	
Entirely public	858 (86.1)
Private/insurance/mixed	139 (13.9)
Body Mass Index at enrolment (kg/m^2^) ^a^	
Underweight (< 21.5)	173 (17.4)
Normal weight (21.5–26.2)	389 (39.0)
Overweight (26.3–30.9)	262 (26.3)
Obesity (> 30.9)	172 (17.3)
Smoking	
No smoking	923 (92.6)
Smoking (at any time of pregnancy	74 (7.4)
Alcohol drinking ^b^	
No alcohol	722 (72.4)
Drinker	147 (14.7)
Using Other Drugs ^c^	44 (4.4)
Maternal pre-existing conditions ^e^	124 (12.4)
**Total**	997

Missing information for a) 1, b) 128, c) 144, d) 137

e) Maternal conditions included anemia, depression, hypertension without use of medication

BMI cut-off values at 19–21 weeks of gestational age according to Atalah et al. [32]

**Table 2:** Pregnancy outcomes of the study population

Pregnancy outcomes	Study population Mean (± SD) or N (%)
Gestation at delivery (weeks)	39.0 (± 1.1)
Birth weight (grams)	3,253 (± 419)
Preeclampsia	64 (6.4)
HIP	127 (12.7)
All caesarean section	465 (46.6)
Apgar < 7 at 5 min ^a^	8 (0.8)
Need for intubation after birth ^b^	3 (0.3)
Need for NICU admission	90 (9.0)
Need for Phototherapy ^b^	147 (14.8)
Neonatal hypoglycemia ^c^	7 (0.7)
Composite of neonatal morbidity	35 (3.6)
Fetal death	0.0
Neonatal death	0.0
**Total**	997

Abbreviations: *NICU* Neonatal intensive care unit, *HIP* Hyperglycemia in pregnancy

Missing information for a) 50, b) 8, c) 1

Composite of neonatal morbidity: at least one of the following conditions: hypoglycemia, seizure, persistent pulmonary hypertension, bronchopulmonary dysplasia, hypoxic-ischemic encephalopathy, intraventricular haemorrhage, necrotizing enterocolitis, sepsis, mechanical ventilation, or oxygen therapy requirement

**Fig. 2:**
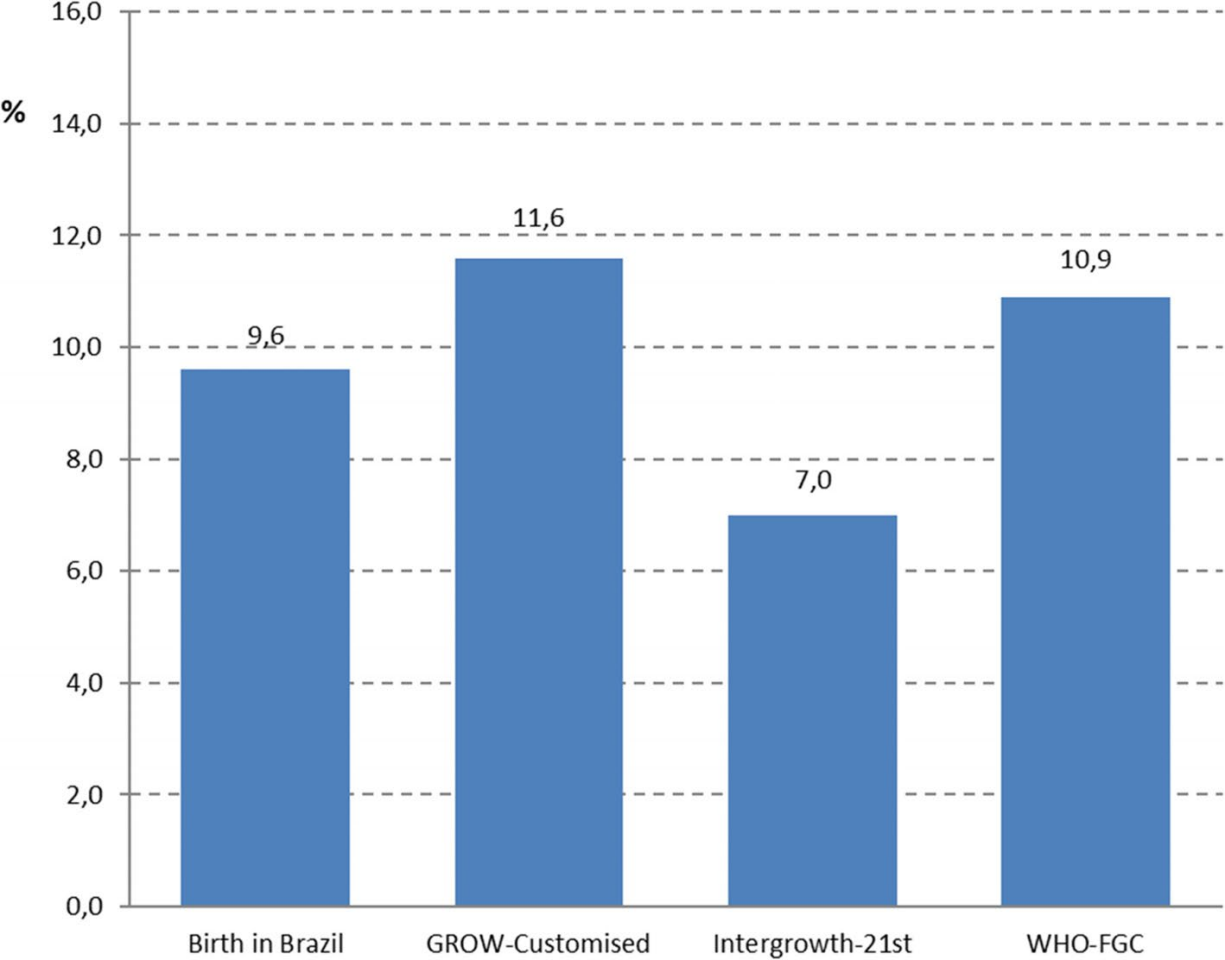
Proportion of nullipara women who gave birth to SGA infants (birthweight < 10^th^ centile) according to different charts

The proportion of infants classified as SGA according to each chart is illustrated in Fig. 2. This proportion ranged from 7.0% using the Intergrowth-21^st^ chart to 11.6% using the GROW-customised chart.

**Fig. 3:**
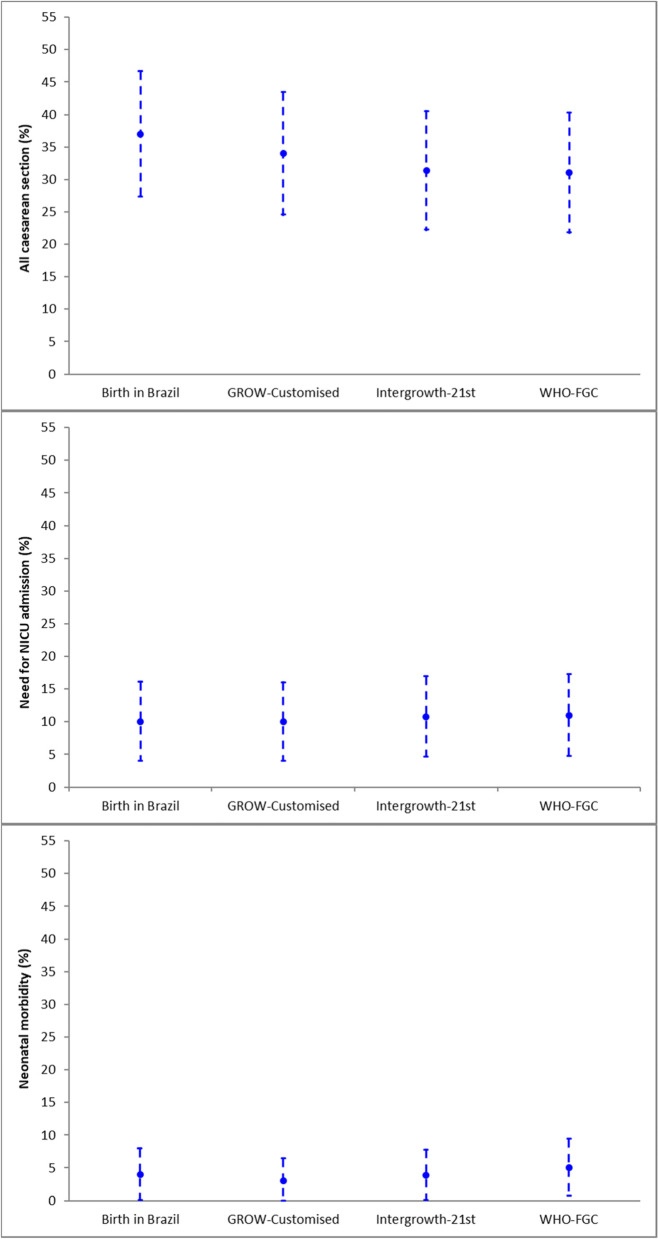
Rate (95% CI) of adverse outcomes up to the 10^th^ birthweight centile according to different charts

In the population of birthweights below the 10^th^ centile, the maternal and neonatal outcome rates according to different charts with their respective 95% confidence intervals are shown in Fig. 3. Neonatal morbidity rates were similar for infants below the 10^th^ centile despite the use of different charts, as were NICU admission rates. In the included population from the Preterm SAMBA study, there were no perinatal mortality cases.

The prevalence of outcomes among neonates with a birth weight below the 5^th^, 10^th^ and 15^th^ percentiles were compared with the AGA neonates with a birth weight between the 40^th^ and 60^th^ percentiles. The Odds Ratios are shown in Table 3. All charts showed a significantly decreased risk of overall caesarean section and the size of the effect varied according to the considered cutoff point of birthweight centile. With regards to neonatal morbidity, there appeared to be an increased risk inversely related to birth weight among SGA neonates according to almost all charts, but the increase in risk was not statistically significant.

**Table 3:** Odds Ratio for maternal and neonatal outcomes among SGA neonates and their mothers (assuming different centile thresholds) compared with the reference group with a birth weight between 40^th^-60^th^ centiles according to each chart

Adverse outcomes and centile thresholds	Birth in Brazil OR (95%CI)	GROW-Customised OR (95%CI)	Intergrowth-21^st^ OR (95%CI)	WHO-FGC OR (95%CI)
**All caesarean section**				
< p5	**0.41 [0.21 – 0.81]**	0.56 [0.30 – 1.06]	0.51 [0.27 – 1.00]	**0.47 [0.24 – 0.93]**
< p10	0.62 [0.38 – 1.02]	**0.53 [0.32 – 0.87]**	**0.57 [0.34 – 0.93]**	**0.56 [0.34 – 0.93]**
< p15	**0.61 [0.40 – 0.95]**	**0.55 [0.35 – 0.84]**	**0.55 [0.35 – 0.85]**	**0.64 [0.41 – 0.99]**
**Need for NICU admission**				
< p5	1.24 [0.47 – 3.27]	1.26 [0.44 – 3.61]	1.34 [0.50 – 3.57]	1.83 [0.71 – 4.72]
< p10	1.01 [0.45 – 2.25]	1.28 [0.56 – 2.94]	1.22 [0.55 – 2.68]	1.42 [0.63 – 3.19]
< p15	1.01 [0.50 – 2.05]	1.19 [0.56 – 2.52]	1.04 [0.50 – 2.15]	1.08 [0.50 – 2.33]
**Neonatal morbidity**				
< p5	1.37 [0.36 – 5.25]	1.13 [0.23 – 5.62]	0.97 [0.20 – 4.74]	1.32 [0.26 – 6.74]
< p10	0.89 [0.27 – 2.98]	0.86 [0.22 – 3.39]	0.97 [0.29 – 3.32]	1.70 [0.51 – 5.72]
< p15	0.74 [0.24 – 2.25]	0.76 [0.22 – 2.64]	0.82 [0.26 – 2.57]	1.11 [0.33 – 3.70]

Abbreviations: *CI* Confidence interval, *NICU* Neonatal intensive care unit, *OR* Odds ratio. Values in bold are statistically significant

The diagnostic performance of each chart to detect SGA infants (birthweight below 5^th^, 10^th^, 15^th^ and 20^th^ centiles) with neonatal morbidity is shown in Table 4. All combinations of thresholds and charts showed a very similar poor performance (AUC 0.492—0.522). The different birthweight centiles demonstrated similar sensitivity and false-positive rate irrespective of the chart. Although the sensitivity roughly doubled from the 10^th^ to the 20^th^ centile, the false positive rate increased proportionately, which is probably due to the small sample size.

**Table 4:** Diagnostic performance, sensitivity and false positive rate of neonatal morbidity in neonates with birthweight below different centile thresholds using different charts in comparison with the “Birth in Brazil” chart

Charts	Centile thresholds	Number of events	Rate (%)	Sensitivity (95%CI)	FPR	AUC
Birth in Brazil						
	5^th^	3	6.0	8.57	4.89	0.518
	**10**^**th**^	4	4.0	11.43	9.98	0.507
	15^th^	5	3.0	14.29	15.07	0.496
	**20**^**th**^	6	3.0	17.14	20.06	0.485
GROW-Customised						
	5^th^	2	3.9	5.71	5.09	0.503
	**10**^**th**^	3	3.0	8.57	10.08	0.492
	15^th^	4	2.7	11.43	15.18	0.481
	**20**^**th**^	6	3.0	17.14	20.17	0.485
Intergrowth-21^st^						
	5^th^	2	3.9	5.71	5.09	0.503
	**10**^**th**^	4	3.9	11.43	10.19	0.506
	15^th^	5	3.3	14.29	15.07	0.496
	**20**^**th**^	6	3.0	17.14	20.17	0.485
WHO-FGC						
	5^th^	2	3.9	5.71	5.09	0.503
	**10**^**th**^	5	5.0	14.29	9.88	0.522
	15^th^	5	3.3	14.29	15.18	0.496
	**20**^**th**^	6	3.0	17.14	20.27	0.484

Abbreviations: *AUC* Area under ROC curve, *FPR* False positive rate, *WHO* World Health Organization, *FGC* Fetal Growth Charts

## Discussion

According to our results, the birthweight charts from Birth in Brazil, Intergowth-21^st^, WHO-FGC and the GROW-customised chart performed similarly for the detection of neonatal morbidity among neonates who are below the considered cutoff centiles. Furthermore, the diagnostic performance of these charts was considered poor given the low AUC values regardless of the different cut-off points of birthweight centiles.

By definition, SGA neonates (birth weight below the 10^th^ centile) are expected to comprise 10% of the population. Given our study population comprised only nulliparous women, it would be expected a prevalence of SGA above 10%. We observed different SGA rates for the studies charts and the Intergrowth-21^st^ chart showed the lowest prevalence rate (7.0%). Based on this finding, it would be reasonable to question if the Intergrowth-21^st^ charts appropriately reflect the Brazilian population. A previous study has similarly found that the Intergrowth-21^st^ birthweight chart detects fewer SGA neonates when compared to GROW customised chart [21] and also when compared to the WHO charts [22]. This indicates that our findings are not specific to a Brazilian population, but rather a common finding in other populations.

Published literature has frequently shown that population and customised charts have different detection rates for SGA as they are made from different methodological approaches [14, 24, 25]. Although the Brazilian population had been included in the studies for the elaboration of international standard population charts, even those charts differed from each other according to the detection rates of SGA when applied to the same population. This was consistent with observations in other Latin-American populations [23].

However, when analyzing the association between those SGA neonates and neonatal morbidity, all charts performed similarly and poorly. In addition, lack of assessment of other demographic factors influencing fetal/infant growth prevents us from drawing any conclusion; Population-based studies are needed to address which birthweight chart is more appropriate in the Brazilian setting.

Nulliparous women tend to have lower levels of serum glucose and worse hemodynamic adaptations than multiparous women during pregnancy [26–28]. These differences may contribute to the higher incidence of SGA neonates born to nulliparous women. A possible reason for the poor accuracy in identifying SGA neonates with adverse outcomes born to nulliparous women is that although this population is associated with a higher risk of SGA [4], those neonates may comprise both constitutionally small and growth-restricted infants. Nulliparous women may be more likely to have constitutionally small infants with mild/no increase in risk of neonatal morbidity [29]. Another reason for such poor performance is that birthweight alone may not be the most reliable predictor of adverse outcomes in SGA. In addition, SGA related adverse outcomes may involve milder conditions that were not fully considered in our composite of neonatal morbidity.

In our study, the composite outcome was developed as a proxy of the composite of hypoxic-ischemic events considered by Chauhan et al [20]. as they have shown the risk of a hypoxic composite was 44% higher in SGA neonates. Although a trend of the increasing size of effects was observed in smaller infants, our results failed to show a statistical increase in risk. Based on the width of the confidence interval, it was likely underpowered to confirm this association. It is also possible that the composite outcome does not accurately represent SGA-related neonatal morbidity among lower-risk nulliparous women.

In a study with a Swedish population comparing different birthweight charts including the Intergrowth-21^st^, GROW-customised charts, and a population reference, the performance of all charts was largely similar and better to detect perinatal mortality than neonatal morbidity after fixing the false-positive rate by 10% [13]. In our analysis, perinatal mortality could not be evaluated since our study found no perinatal mortality cases. A larger sample size would be necessary to obtain sufficient power to evaluate perinatal mortality, which is a very uncommon outcome. Similarly, our sample size did not have enough power to properly evaluate the outcome of a low 5-min Apgar score, since there were only eight cases (0.8%) recorded.

The decreased risk of overall caesarean sections in women giving birth to SGA neonates compared to women with adequate-for-gestational-age (AGA) neonates may be intriguing, especially in Brazil, a country with such high rates of caesarean sections as showed in the outcome rates in Table 2 with a cesarean rate of 46%. It is true that the largest proportion of fetal growth restriction occurs among SGA infants and thereby the risk of caesarean section is expected to be higher in this group when compared to the AGA group. The rationale is based on the higher risk of non-reassuring fetal status among cases of FGR [30]. It is possible that the main drivers for caesarean section rates in Brazil are other factors than non-reassuring fetal status. These other factors may act as negative confounders for the association between SGA and CS. Indeed, published data have shown that caesarean section rates increase in direct proportion to birthweight in Brazil [31].

This study has limitations for being a secondary analysis of data collected for different purposes other than detecting SGA rates and adverse perinatal outcomes related to hypoxic-ischemic morbidity. By restricting the subjects to neonates born at term, the sample became such that the power was limited to adequately evaluate important adverse outcomes such as perinatal mortality and low 5-min Apgar. Also, this study did not evaluate long-term adverse outcomes related to SGA. The strength of this study is that it evaluates SGA among a specific population of nulliparous women and compares the performances of the most used birthweight charts (population and customised charts) in a Brazilian population of lower-risk pregnant women.

## Conclusions

The population charts from Birth in Brazil, Intergrowth-21^st^ and WHO-FGC and the GROW-customised chart showed a similar and poor performance to identify neonatal morbidity related to SGA neonates. To detect adverse outcomes more accurately, further studies are needed with sufficient statistical power to evaluate important hypoxic-related outcomes such as low 5-min Apgar scores and perinatal mortality rates among newborns from nulliparous women.

## Supplementary Information

**Additional file 1.**

## Data Availability

The dataset used and analysed during the current study is available from the corresponding author upon reasonable request.

## Abbreviations

AGA: Adequate for Gestational Age
AUC: Area Under the Curve
FGR: Fetal Growth Restriction
GROW: Gestation Related Optimal Weight
NICU: Neonatal Intensive Care Unit
ROC: Receiver Operating Characteristic
SGA: Small for Gestational Age
WHO-FGC: World Health Organization – Fetal Growth Charts

## References

[1] Nardozza LM, Caetano AC, Zamarian AC, Mazzola JB, Silva CP, Marçal VM (2017). Fetal growth restriction: current knowledge. Arch Gynecol Obstet.

[2] Melamed N, Baschat A, Yinon Y, Athanasiadis A, Mecacci F, Figueras F (2021). FIGO (international Federation of Gynecology and obstetrics) initiative on fetal growth: best practice advice for screening, diagnosis, and management of fetal growth restriction. Int J Gynaecol Obstet..

[3] Lees CC, Stampalija T, Baschat A, da Silva CF, Ferrazzi E, Figueras F (2020). ISUOG Practice Guidelines: diagnosis and management of small-for-gestational-age fetus and fetal growth restriction. Ultrasound Obstet Gynecol.

[4] Souza RT, Vieira MC, Esteves-Pereira AP, Domingues RMSM, Moreira MEL, Filho EVC (2020). Risk stratification for small for gestational age for the Brazilian population: a secondary analysis of the Birth in Brazil study. Sci Rep..

[5] Figueras F, Gratacos E (2014). Stage-based approach to the management of fetal growth restriction. Prenat Diagn.

[6] Beune IM, Bloomfield FH, Ganzevoort W, Embleton ND, Rozance PJ, van Wassenaer-Leemhuis AG (2018). Consensus Based Definition of Growth Restriction in the Newborn. J Pediatr.

[7] Gordijn SJ, Beune IM, Thilaganathan B, Papageorghiou A, Baschat AA, Baker PN (2016). Consensus definition of fetal growth restriction: a Delphi procedure. Ultrasound Obstet Gynecol.

[8] Kiserud T, Piaggio G, Carroli G, Widmer M, Carvalho J, Neerup Jensen L (2017). The World Health Organization Fetal Growth Charts: A Multinational Longitudinal Study of Ultrasound Biometric Measurements and Estimated Fetal Weight. PLoS Med.

[9] Villar J, Cheikh Ismail L, Victora CG, Ohuma EO, Bertino E, Altman DG (2014). International standards for newborn weight, length, and head circumference by gestational age and sex: the Newborn Cross-Sectional Study of the INTERGROWTH-21st Project. Lancet.

[10] Souza RT, Vieira MC, Esteves-Pereira AP, Domingues R, Moreira MEL, da Cunha Filho EV (2020). Risk stratification for small for gestational age for the Brazilian population: a secondary analysis of the Birth in Brazil study. Sci Rep.

[11] Gardosi J, Francis A, Turner S, Williams M (2018). Customized growth charts: rationale, validation and clinical benefits. Am J Obstet Gynecol.

[12] Gardosi J (2004). Customized fetal growth standards: rationale and clinical application. Semin Perinatol.

[13] Vieira MC, Relph S, Persson M, Seed PT, Pasupathy D (2019). Determination of birth-weight centile thresholds associated with adverse perinatal outcomes using population, customised, and Intergrowth charts: A Swedish population-based cohort study. PLoS Med.

[14] Cartwright RD, Anderson NH, Sadler LC, Harding JE, McCowan LME, McKinlay CJD (2020). Neonatal morbidity and small and large size for gestation: a comparison of birthweight centiles. J Perinatol.

[15] Cecatti JG, Souza RT, Sulek K, Costa ML, Kenny LC, McCowan LM (2016). Use of metabolomics for the identification and validation of clinical biomarkers for preterm birth: Preterm SAMBA. BMC Pregnancy Childbirth.

[16] Souza RT, Cecatti JG, Costa ML, Mayrink J, Pacagnella RC, Passini R (2019). Planning, Implementing, and Running a Multicentre Preterm Birth Study with Biobank Resources in Brazil: The Preterm SAMBA Study. Biomed Res Int.

[17] Souza RT, Costa ML, Mayrink J, Feitosa FE, Rocha Filho EA, Leite DF (2020). Perinatal outcomes from preterm and early term births in a multicenter cohort of low risk nulliparous women. Sci Rep.

[18] Mayrink J, Souza RT, Feitosa FE, Rocha Filho EA, Leite DF, Vettorazzi J (2019). Incidence and risk factors for Preeclampsia in a cohort of healthy nulliparous pregnant women: a nested case-control study. Sci Rep.

[19] Nicolosi BF, Souza RT, Mayrink J, Feitosa FE, Rocha Filho EA, Leite DF (2020). Incidence and risk factors for hyperglycemia in pregnancy among nulliparous women: A Brazilian multicenter cohort study. PLoS ONE.

[20] Chauhan SP, Rice MM, Grobman WA, Bailit J, Reddy UM, Wapner RJ (2017). Neonatal Morbidity of Small- and Large-for-Gestational-Age Neonates Born at Term in Uncomplicated Pregnancies. Obstet Gynecol.

[21] Pritchard N, Lindquist A, Siqueira IDA, Walker SP, Permezel M (2020). INTERGROWTH-21st compared with GROW customized centiles in the detection of adverse perinatal outcomes at term. J Matern Fetal Neonatal Med.

[22] Choi SKY, Gordon A, Hilder L, Henry A, Hyett JA, Brew BK (2021). Performance of six birth-weight and estimated-fetal-weight standards for predicting adverse perinatal outcome: a 10-year nationwide population-based study. Ultrasound Obstet Gynecol.

[23] SI L, B H, O Eo, CB M (2021). Maternal Risk Factors for Small-for-Gestational-Age Newborns in Mexico: Analysis of a Nationwide Representative Cohort. Front Public Health..

[24] Mendez-Figueroa H, Chauhan SP, Barrett T, Truong VTT, Pedroza C, Blackwell SC (2019). Population versus Customized Growth Curves: Prediction of Composite Neonatal Morbidity. Am J Perinatol.

[25] Francis A, Hugh O, Gardosi J (2018). Customized vs INTERGROWTH-21(st) standards for the assessment of birthweight and stillbirth risk at term. Am J Obstet Gynecol.

[26] Eldin Ahmed Abdelsalam K, Alobeid MEA (2017). Influence of Grand Multiparity on the Levels of Insulin, Glucose and HOMA-IR in Comparison with Nulliparity and Primiparity. Pak J Biol Sci..

[27] Ling HZ, Guy GP, Bisquera A, Poon LC, Nicolaides KH, Kametas NA (2019). The effect of parity on longitudinal maternal hemodynamics. Am J Obstet Gynecol..

[28] Wright E, Audette MC, Ye XY, Keating S, Hoffman B, Lye SJ (2017). Maternal Vascular Malperfusion and Adverse Perinatal Outcomes in Low-Risk Nulliparous Women. Obstet Gynecol.

[29] Ananth CV, Vintzileos AM (2009). Distinguishing pathological from constitutional small for gestational age births in population-based studies. Early Hum Dev.

[30] Smith GC (2000). A population study of birthweight and the risk of caesarean section: Scotland 1980–1996. BJOG.

[31] Gomes UA, Silva AA, Bettiol H, Barbieri MA (1999). Risk factors for the increasing caesarean section rate in Southeast Brazil: a comparison of two birth cohorts, 1978–1979 and 1994. Int J Epidemiol.

[32] Atalah E, Castillo C, Castro R, Aldea A (1997). Proposal of a new standard for the nutritional assessment of pregnant women. Rev Med Chil.

