# Supplementary material for: Performances of birthweight charts to predict adverse perinatal outcomes related to SGA in a cohort of nulliparas
Source: BMC Pregnancy Childbirth. 2022 Aug 4;22:615. doi: 10.1186/s12884-022-04943-1 (PMC9351115; doi:10.1186/s12884-022-04943-1)
Supplement: Supplementary file 1 — Additional file 1. [file 12884_2022_4943_MOESM1_ESM.docx]

Supplementary file S1

**S1. Preterm SAMBA study group consortium**

Rafael B Galvão^1^, Renato T Souza^1^, Matias C Vieira^1,2^, Dharmintra Pasupathy^2,3^, Jussara Mayrink^1^, Francisco E. Feitosa^4^, Edilberto A Rocha Filho^5^, Débora F Leite^5^, Janete Vettorazzi^6^, Iracema M Calderon^7^, Maria H Sousa^8^, Jose G. Cecatti^1*^, Maria Laura Costa^1^, Rodolfo C Pacagnella^1^, Renato Passini Jr^1^, Mary A. Parpinelli^1^, Karayna G Fernandes^1,8^, José Paulo Guida^1^, Danielly S Santana^1^, Bianca F. Cassettari^7^, Lucia Pfitscher^6^, Luiza Brust^6^, Elias F Melo Junior^5^, Danilo Anacleto^5^, Daisy Pinheiro^4^, Benedita Souza^4^
